# A-Kinase Anchoring Protein 1: Emerging Roles in Regulating Mitochondrial Form and Function in Health and Disease

**DOI:** 10.3390/cells9020298

**Published:** 2020-01-26

**Authors:** Yujia Liu, Ronald A. Merrill, Stefan Strack

**Affiliations:** Department of Neuroscience and Pharmacology, Iowa Neuroscience Institute, University of Iowa Carver College of Medicine, Iowa City, IA 52242, USA; yujia-liu@uiowa.edu (Y.L.); ronald-merrill@uiowa.edu (R.A.M.)

**Keywords:** AKAP1, PKA, Drp1, metabolism, mitochondrial dysfunction, mitochondrial fission, heart failure, cancer, neurodegeneration

## Abstract

Best known as the powerhouse of the cell, mitochondria have many other important functions such as buffering intracellular calcium and reactive oxygen species levels, initiating apoptosis and supporting cell proliferation and survival. Mitochondria are also dynamic organelles that are constantly undergoing fission and fusion to meet specific functional needs. These processes and functions are regulated by intracellular signaling at the mitochondria. A-kinase anchoring protein 1 (AKAP1) is a scaffold protein that recruits protein kinase A (PKA), other signaling proteins, as well as RNA to the outer mitochondrial membrane. Hence, AKAP1 can be considered a mitochondrial signaling hub. In this review, we discuss what is currently known about AKAP1′s function in health and diseases. We focus on the recent literature on AKAP1′s roles in metabolic homeostasis, cancer and cardiovascular and neurodegenerative diseases. In healthy tissues, AKAP1 has been shown to be important for driving mitochondrial respiration during exercise and for mitochondrial DNA replication and quality control. Several recent in vivo studies using AKAP1 knockout mice have elucidated the role of AKAP1 in supporting cardiovascular, lung and neuronal cell survival in the stressful post-ischemic environment. In addition, we discuss the unique involvement of AKAP1 in cancer tumor growth, metastasis and resistance to chemotherapy. Collectively, the data indicate that AKAP1 promotes cell survival throug regulating mitochondrial form and function. Lastly, we discuss the potential of targeting of AKAP1 for therapy of various disorders.

## 1. Introduction

### 1.1. The cAMP/PKA Axis and AKAPs

Signal transduction downstream of growth factors, hormones, and neurotransmitters engages second messengers including cyclic adenosine monophosphate (cAMP). Since Earl W. Sutherland’s discovery of cAMP in 1958 [1,2], for which he was awarded the 1971 Nobel Prize in Physiology or Medicine, the second messenger has been implicated in a multitude of cellular processes, including growth, differentiation, and survival. cAMP is synthesized from ATP by a family of adenylyl cyclases, which are regulated by heterotrimeric G proteins or Ca^2+^/calmodulin in response to cell surface receptor signaling. cAMP actions are mediated by two effectors, cAMP-dependent protein kinase (PKA) and the more recently discovered guanylate exchange factor Epac (exchange protein directly activated by cAMP) [3]. cAMP signaling is terminated by the hydrolysis of cAMP to 5′-AMP by a large group of phosphodiesterases, which are themselves highly regulated [4]. Activation of PKA by cAMP is well understood and has been reviewed recently [5]. When cellular cAMP concentrations are low, PKA exists as an inactive tetramer composed of two regulatory subunits and two catalytic subunits. Upon binding of cAMP, the two catalytic subunits are then released to phosphorylate PKA substrates, eliciting downstream signaling. A kinase anchoring proteins (AKAPs) contribute specificity to PKA signaling by recruiting the kinase to different locations within the cell. AKAPs are a group of structurally unrelated proteins that have in common the ability to bind PKA regulatory subunits with high affinity [6,7]. Because AKAPs interact with regulatory, rather than catalytic subunits of PKA, initiation of substrate phosphorylation still requires cAMP-mediated release of the active catalytic subunit for compartmentalized signaling within the cell. The generation of cAMP and its degradation by phosphodiesterases at the surface and inner compartments of the mitochondria has recently been reviewed [8,9].

### 1.2. Discovery of AKAP1

In the early 1990s, the first group of AKAPs was discovered by John Scott ‘s team as functionally diverse polypeptides that compartmentalized PKA within the cell [10,11]. AKAP1 was identified in 1995 as S-AKAP84 in mammalian spermatozoa by Charles Rubin’s group. Interestingly, S-AKAP84 was found to be a PKA anchoring protein with an N-terminal mitochondrial targeting domain, and the group discovered that S-AKAP84 localization closely correlates with the localization of mitochondria during sperm development. The anchoring protein follows mitochondrial accumulation as the spermatids undergo nuclear condensation and tail elongation, processes that require high levels of energy [12]. Subsequently in 1996, human AKAP149 was discovered as a splice variant of S-AKAP84. Christoph Hanski and colleagues also described its C-terminal mRNA binding KH–Tudor domains [13]. Rubin’s group then cloned the murine S-AKAP84 cDNA and identified several of its isoforms in 1997, including a long form which they named AKAP121 based on its molecular mass [14]. In the same year, Susan Taylor’s group discovered and characterized the binding of AKAP121 to both RI and RII regulatory subunits of PKA, thereby identifying AKAP121 as dual-specificity AKAP1, also referred as D-AKAP1 [15]. Taylor’s group later suggested that murine AKAP1 can be differentially targeted to endoplasmic reticulum (ER) or mitochondria by alternative splicing of the N-terminus [16,17,18]; however, ER-targeted AKAP1 isoforms do not exist in other species, such as rats and humans [19]. Mitochondrial localization is critical for AKAP1′s function, as removal of the N-terminal mitochondrial targeting sequence from the full-length protein diminishes its effects [20,21]. Other isoforms of AKAP1 have also been discovered by genomic and biochemical methods; however, we will be focusing on the most abundant, full-length form of AKAP1 in this review.

### 1.3. Overview of AKAP1: Role in Mitochondrial Fission and Fusion and Beyond

**Figure 1:**
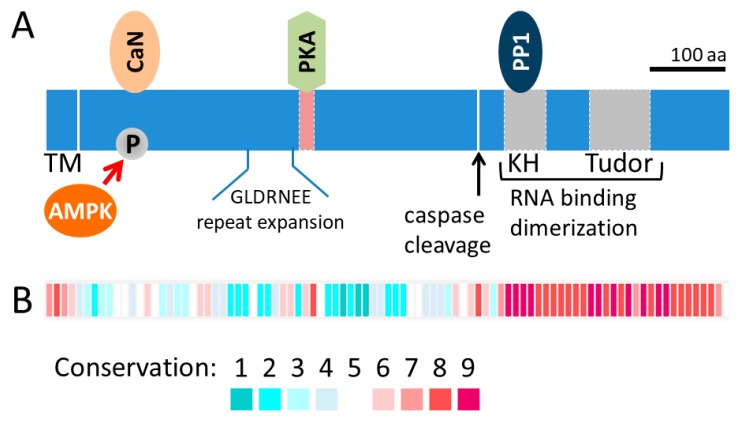
AKAP1 protein domain structure and sequence conservation. (**A**) Protein domain schematic of AKAP1 with the N-terminal OMM transmembrane at the left and the KH and Tudor domains at the C-terminus on the right. Included are some of the interacting proteins, CaN, PKA and PP1, and sites of post-translational modification by AMPK and caspases. The GLDRNEE repeat is expanded in many mammals, including humans, and it accounts for some of the variation in molecular weights across species. (**B**) Blocks represent the average conservation score of 10 residues and correspond to the diagram in (A). Block conservation was determined from the alignment of 113 orthologs using the ConSurf web server (http://consurf.tau.ac.il/). Abbreviations: AMPK, 5’ adenosine monophosphate-activated protein kinase; CaN, calcineurin; KH, K homology; PKA, protein kinase A; PP1, protein phosphatase 1; TM, transmembrane.

Research over the past several decades has revealed the important role of mitochondria in cellular signaling involving proliferation, differentiation and survival. AKAP1 was discovered as a mitochondrial PKA anchor with a mitochondrial targeting sequence that is inserted into the outer mitochondrial membrane (OMM) and the remaining protein residing in the cytosol (Figure 1a). Indeed, the first 30 residues of AKAP1 are sufficient to recruit other proteins, such as GFP, to the OMM [18]. AKAP1 has no known enzymatic activity, but instead is a scaffolding protein that acts as a signaling hub at the OMM [19]. The protein sequence conservation for the first coding exon, which encodes the majority of the protein, is low even amongst mammals (Figure 1b). For example, the human AKAP1 protein sequence is only 52% identical to the mouse sequence across the first coding exon (protein blast of human NP_001229831 [amino acids 1–571] compared to mouse NP_033778 [amino acids 1–525]). Due to variation in the protein size across species and additional splice-variants, older literature may refer to the protein by names other than the HUGO name (e.g., D-AKAP1, AKAP121, AKAP149, AKAP84).

AKAP1 has been reported to interact with more than twenty proteins and mRNAs, which was the focus of a previous review [19]. More recent work on AKAP1 has examined expression levels and the influence of post-translational modifications on AKAP1′s function. The AKAP1 knockout mouse was generated by deletion of the first coding exon that includes the mitochondrial targeting sequence and the PKA binding site [22]. Even though the AKAP1 knockout mouse was generated over a decade ago [22], a recent flurry of publications has described the use of the AKAP1 gene deletion to tease out the function of AKAP1 in a variety of tissues [23,24,25,26,27,28]. As we begin to discuss the specific roles of AKAP1 in mitochondrial form and function, it is worth noting how the dynamic changes in mitochondrial morphology impact function. Mitochondria form a dynamic network molded by opposing fission and fusion events. Mitochondrial fission is catalyzed by dynamin-related protein 1 (Drp1) located at the OMM while mitochondrial fusion is mediated by optic atrophy 1 (Opa1) and mitofusin 1/2, which are responsible for fusion of the inner and outer mitochondrial membrane, respectively [29,30]. In recent years, researchers have revealed numerous functions of mitochondria beyond their most well-known role in ATP production, including roles in protein synthesis, reactive oxygen species (ROS) and Ca^2+^ handling, cell survival and more [31]. Studies on how the dynamic changes of mitochondrial shape modulates function reveal that mitochondrial fission can be important for their transport and apoptosis, while mitochondrial fusion increases cell respiratory capacity, helping cells in stressful environments [32]. This review will focus on the most recent in vivo reports on the impact of AKAP1 in mitochondrial form and function, as well as on AKAP1 expression and posttranslational modification in health and various diseases.

## 2. Mitochondrial AKAP1 in Physiological Conditions

### 2.1. Metabolic Homeostasis

Nuclear receptor peroxisome proliferator-activated receptor γ (PPARγ) is a major regulator of energy metabolism [33]. AKAP1 has been shown to be a direct target of transcription factor PPARγ in the regulation of lipid metabolism [19,34]. Exercise plays important roles in modulating metabolic homeostasis and maintaining insulin sensitivity. Increases in physical activity initiate a systemic metabolic challenge throughout the body, leading to whole-body glucose and lipid metabolism and improvements in insulin sensitivity [35]. AMP-activated protein kinase (AMPK) is a key enzyme in energy homeostasis. In fact, both PKA and AMPK feature prominently in muscle exercise signaling [36]. In a recent study comparing the phosphoproteome from human muscle biopsies following acute exercise, several AMPK substrates were identified, including AKAP1. Following AMPK activation, phosphorylation of S103 of rat AKAP1 (corresponding to human S107) was shown to be necessary for the AMPK-induced increases in mitochondrial respiration [37]. However, the underlying mechanism by which AMP-mediated phosphorylation of AKAP1 led to an increase in mitochondrial respiration was not determined.

### 2.2. Mitochondrial Quality Control

Because ROS is a byproduct of mitochondrial ATP production, integrated cellular signaling pathways modulate the turnover of damaged organelles to maintain a functional mitochondrial network [38]. Several recent studies have examined the role of AKAP1 in mitochondrial quality control. MDI, the AKAP1 ortholog in *Drosophila*, has been shown to be essential for mitochondrial (mt)DNA replication and translation of nuclear-encoded mitochondrial proteins at the OMM. MDI deletions in the mitochondrial targeting sequence, the PKA regulator subunit binding sequence or the TUDOR domain reduced mtDNA replication and localized mitochondrial protein translation. MDI binding to the translation stimulator La-related protein (Larp) appears to be critical for this effect, as overexpressing an artificially mitochondria-targeted Larp could rescue the phenotypes of the MDI-deficient flies. This indicates that stimulation of local protein synthesis is an essential function of MDI. The expression of the human AKAP1 protein could also rescue the MDI loss in *Drosophila* [39]. Since the KH–Tudor domain of mammalian AKAP1 is known to bind mRNAs that encode mitochondrial proteins [40,41,42], fostering localized protein synthesis may be a conserved function of AKAP1. A follow-up study from the same group further showed that MDI plays important roles in mediating proper PTEN-induced kinase 1 (PINK1) signaling for initiation of mitophagy [43]. In heteroplasmic oocytes with a normal and a mutated form of mtDNA, subcellular localization analysis confirmed PINK1 accumulation on mitochondria that carried the mutant mtDNA.

**Figure 2:**
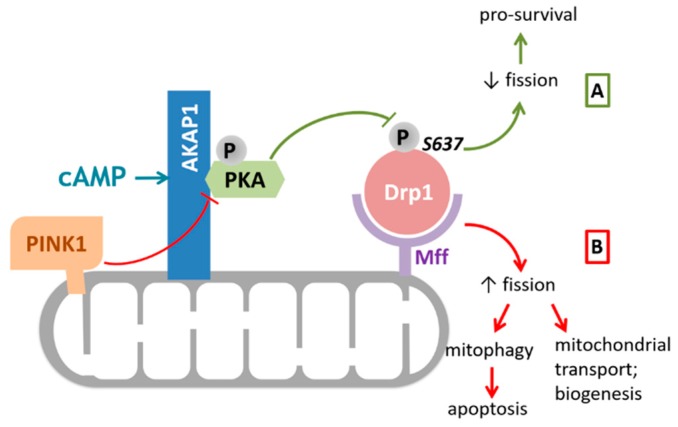
AKAP1 modulates mitochondrial fission by regulating Drp1 activity via PKA. PKA can be recruited to the OMM by binding to AKAP1. (**A**) Drp1 is recruited to the mitochondria via Mff where, upon activation by cAMP, PKA deactivates Drp1 through phosphorylation at Ser637. The resulting opposed mitochondrial fission promotes cell survival under pathological conditions such as ischemia and reperfusion injury. (**B**) PINK1 can dissociate the AKAP1–PKA complex in a kinase activity dependent manner and therefore blocks the anti-fission role of AKAP1–PKA. Increased mitochondrial fission through unopposed Drp1 activation is important for the initiation of mitophagy and apoptosis, as well as for mitochondrial biogenesis and transport. Abbreviations: AKAP1, A kinase anchoring protein 1; Drp1, dynamin related protein 1; cAMP, cyclic adenosine monophosphate; Mff, mitochondrial fission factor; PKA, protein kinase A; PINK1, PTEN-induced kinase 1.

Moreover, Pryde et al. showed that controlled OMM localized PINK1 was shown to drive Drp1-dependent mitochondrial fission in a catalytic activity-dependent manner, presumably by directly phosphorylating AKAP1 and disrupting the AKAP1–PKA association [44] (Figure 2). This finding indicates that PINK1 modulation of the AKAP1–PKA signaling axis selectively promotes Drp1-mediated fission of damaged mitochondria, thus ensuring initiation of mitophagy in mammalian cells [44].

## 3. Mitochondrial AKAP1 in Disease Pathogenesis

### 3.1. AKAP1 in Cardiovascular Diseases

Cardiomyocytes rely on proper mitochondrial function to support the high-energy demand required for contraction. Mitochondria are critical for cell survival in acute injuries to the heart and lungs, in part due to their ability to regulate cellular ROS generation and sequestration [27,45,46]. Therefore, AKAP1 is considered a cardioprotective scaffolding protein of PKA. Several recent reports discussed below collectively support the important role of AKAP1 in cardiovascular health and also elucidate its role in the pathogenesis of cardiac injury.

Upon oxygen-glucose deprivation, the E3-ubiquitin ligase Siah2 (Seven In-Absentia Homolog 2) is induced by HIF1α and binds AKAP1 to mediate its degradation [47]. Hypoxia and myocardial infarction induced mitochondrial fission and cell death are associated with AKAP1 degradation. Genetic deletion of Siah2 prevents AKAP1 protein loss and protects against myocardial infarction induced cell death and loss of heart function [48]. Furthermore, AKAP1 knock-out mice have an increased sensitivity to myocardial infarction that is demonstrated by decreases in heart function and survival. The mechanism appears to be through an increase in mitophagy, which was observed in the AKAP1 KO mice. Treatment with the autophagy inhibitor 3-methyladenine decreased the loss of heart function in AKAP1 KO mice but not in wildtype mice [24].

AKAP1 and Siah2 also play similar antagonistic roles in cardiac hypertrophy. Transverse aortic constriction (TAC) generates heart failure that is exacerbated by AKAP1 gene loss, even in the heterozygous mouse. Siah2 KO mice retain AKAP1 protein expression, compared to wildtype mice, following TAC; however, these mice are not protected against heart failure [25]. AKAP1 has also been studied in the regulation of vascular functions. The same group also reported alterations of endothelial cell behavior in AKAP1 KO mice as well as a mild increase in blood pressure [26].

Impaired cardiac fatty acid metabolism and lipotoxicity contribute to heart failure [49]. Tsushima et al. recently showed that lipid overload accelerates AKAP1 turnover through ubiquitination/proteasome degradation. This observation appears to be independent of the HIF1α induced Siah2 expression of the hypoxia-stimulated pathway. Decreased AKAP1 levels directly reduced PKA localization to the mitochondria, which leads to unopposed mitochondrial fission through decreased inhibitory phosphorylation of Drp1 at S637. The subsequent increase in cytosolic ROS further contributes to cardiac hypertrophy and heart failure [50].

In a model of cardiac ischemia-reperfusion injury, increases in ROS lead to oxidation and loss of the RIα regulatory subunit of PKA. Despite the loss of AKAP1 under this condition, there was an observed increase in the activity of the catalytic subunit of PKA (PKAc), presumably due to loss of the inhibitory RIα subunit. Indeed, overexpression of RIα increased apoptosis following cardiac ischemia/reperfusion injury [28].

In addition to the above-mentioned increases in mitophagy upon AKAP1 deletion under hypoxia conditions, another study demonstrated that hyperoxia conditions also increase mitophagy in AKAP1 KO mice. Following 48 h of hyperoxia treatment, the study found significantly increased PINK1 and Parkin expression in the AKAP1 KO animals, along with elevation of a number of inflammatory cytokines in lung tissue [27]. While this study examined acute lung injury by prolonged exposure to high oxygen concentration and the other studies mentioned above looked at ischemic heart injuries, these studies collectively demonstrate the important role of AKAP1 in preserving mitochondrial functions to promote cell survival under the stressful conditions of both high and low oxygen levels.

Lastly, mitochondrial dysfunction plays a critical role in the pathology of diabetic kidney disease as recently reviewed by Forbes and Thorburn [51]. A recent clinical study reported that AKAP1 expression is elevated in kidney samples from patients suffering from diabetic nephropathy [52]. The authors reported elevated mitochondrial fission in the podocytes in patients with diabetic nephropathy despite also seeing an increased expression of AKAP1. However, the authors did not look further into the mechanisms underlying this increase in AKAP1 expression or discuss the implications of this findings [52]. Whether this finding is unique to podocytes or diabetic nephropathy will need to be revealed in future studies.

### 3.2. AKAP1 in Cancer Progression and Treatment

Rapidly replicating cancer cells have a high energy demand. The roles of mitochondria in cancer have been studied extensively over the past decades, especially the Warburg Effect of cancer cells preferring glycolysis over oxidative phosphorylation [53,54,55]. These studies have led to interest in targeting mitochondrial functions to develop novel cancer therapies. Several recent studies have suggested that the role of AKAP1 in cancer is complicated due to the heterogeneous nature of cancer cells in different stages of tumor growth and metastasis. While one study suggested AKAP1 inhibits cancer cell invasiveness [56], other studies have reported a poorer prognosis with higher AKAP1 expression [57,58].

AKAP1 was one of fifteen mitochondrial-related markers found to be elevated in epithelial breast cancer cells. In a broader scope, it was suggested that mitochondria of the catabolic tumor stroma are supplying the anabolic epithelial layer with metabolites generated via glycolysis, fueling the upregulated oxidative phosphorylation (OXPHOS) reactions and ultimately promoting tumor growth [54,55,59]. Thus, the role of AKAP1 in regulating mitochondrial form and function in this context is an important area of research. Several studies from other groups published in recent years have also shown higher AKAP1 expression is associated with poor prognosis in different human cancers. One study suggested that AKAP1 can serve as a biomarker for hepatocellular carcinoma with qPCR and immunostaining evidence showing AKAP1 upregulation in tumors along with a correlation with poor prognosis [57]. Another study also showed that AKAP1 overexpression inversely correlates with patient survival in non-small cell lung cancer [58]. The authors identified AKAP1 as a transcriptional target of the oncogene myc. Their data suggested that elevation of AKAP1 in high-grade tumors enhances activation of the mammalian target of the rapamycin (mTOR) pathway by direct interaction with an mTORC1 inhibitor, sestrin2, providing new mechanistic insight into the role of AKAP1 in tumor growth [58]. Interestingly, in a recent report about cisplatin resistance in gastric cancer, the authors discovered that the microRNA miR-148a-3p directly targets AKAP1 and leads to greater Drp1 activation, which sensitizes cancer cells to cisplatin treatment. Higher levels of AKAP1 were indicated as contributing to chemotherapy failure in gastric cancer by preventing P53-mediated mitochondrial fission, thus protecting cancer cells from undergoing apoptosis [60]. The above discussed recent literature collectively shows that AKAP1 promotes the survival and growth of cancer cells.

Conversely, reduction of AKAP1 expression was found to be an indicator of malignancy transition in breast cancer progression. Reduced levels of AKAP1 were observed in invasive breast cancer cell lines, as well as in patient biopsies taken from sites of metastasis [56]. The decreased AKAP1 level is thought to be associated with metabolic reprogramming from oxidative phosphorylation to glycolysis as the cancer cell required faster energy production. Moreover, mitochondrial fission resulting from AKAP1 downregulation may increase the efficiency of mitochondrial transportation toward the high energy demanding leading edge of metastatic cells. Thus, reduced AKAP1 expression can be viewed as a biochemical switch toward increased breast cancer metastasis [56]. While this study may appear to be contradictory to studies where increased AKAP1 has been viewed as a “bad” sign for cancer patients, a closer look opens up very fascinating discussions about the dynamic changes adopted by cancer cells when transitioning at different disease stages.

In summary, we can perhaps picture the following scenario for cancer progression: at the tumor stroma, the cells metabolically favor glycolysis and fuel the epithelial cancer cells, which adopt high OXPHOS cellular respiration (high AKAP1) to promote tumor growth [54,55,59]. During the metastasis stage, cells in the blood stream are bathed in nutrients and therefore do not have a high demand on using OXPHOS, however downregulation of AKAP1 and the associated mitochondria fission and increased mitochondria biogenesis can better serve the needs of tissue invasion [56]. Future studies will be of interest in understanding the differential regulation of AKAP1 in cancer pathogenesis. In summary, when evaluating the potential of using AKAP1 as a diagnostic biomarker or drug target for cancer treatment, the heterogeneity of cancer cell population needs to be taken into serious consideration.

### 3.3. AKAP1 in Neuronal Diseases

Mitochondrial dysfunction contributes to many neurological disorders [61]. Various studies have shown that balanced mitochondrial dynamics play a vital role in maintaining neuronal health and in meeting the diverse needs of highly plastic neuronal networks [31]. While AKAP1 has been shown to interact with several signaling proteins, the most well-established role of AKAP1 is through targeting PKA to the mitochondria. This is particularly important in neurons where increasing AKAP1 expression increases mitochondrial length and membrane potential and decreases sensitivity of cultured neurons to several different cell stresses. On the other hand, decreasing AKAP1 expression has the opposite effects [23].

Our laboratory has previously described the role of the AKAP1/PKA axis in neuroprotection [62]. In a recent report from our laboratory, these findings were extended in vivo when mice lacking AKAP1 were shown to have increased sensitivity to stroke injury. AKAP1 KO mice showed increased infarct volume following middle cerebral ischemia occlusion and reperfusion stress to the affected brain area [23]. Mechanistically, loss of AKAP1 leads to decreased Drp1 phosphorylation by PKA and decreases in complex II function. Loss of AKAP1 increases ROS production and Ca^2+^ deregulation in cultured hippocampal neurons treated with glutamate and is dependent on S637 dephosphorylation [23]. In HT-22 cells, the AKAP1–PKA axis provides protection against glutamate toxicity similarly through phosphorylation and inhibition of Drp1 to maintain normal mitochondrial function [63].

In cultured hippocampal neurons, increasing AKAP1 expression increases dendritic outgrowth but decreases the number of synapses. These effects require both an AKAP1–PKA interaction and phosphorylation of Drp1. Our lab has previously examined phospho-regulation of Drp1 in neuronal morphogenesis and mitochondrial bioenergetics in cultured neurons [64]. Since we know AKAP1 KO mice are more sensitive to stroke, future studies on changes occurring in the neurocircuitry in the AKAP1 KO mice, and perhaps the resulted changes learning and memory on the behavioral level will be very interesting in terms of exploring novel roles of AKAP1.

Disrupted mitochondrial dynamics have been reported in the Alzheimer’s disease mouse model and in amyloid beta (Aβ) accumulated scenarios [65,66,67]. Aβ oligomers have been shown to inhibit the PKA/CREB pathway [68], and activation of PKA in hippocampal neurons can counteract the effects of acute expression of Aβ on mitochondrial trafficking and induction of cell death [69]. One study showed that treatment of an estrogen receptor β (ERβ) agonist resulted in increases in Drp1 S637 phosphorylation and mitochondrial fusion and protection against Aβ in hippocampal neurons. The ERβ interacts with AKAP1 and is localized to the mitochondria [70].

Mutation of PINK1 resulting in loss of activity is the second most frequent cause of autosomal recessive Parkinson’s disease. In cultured cortical neurons lacking PINK1, there is a decrease in neurite outgrowth and mitochondrial trafficking. Overexpression of AKAP1 appears to rescue these deficits through phosphorylation of miro-2. However, the underlying mechanism that connects AKAP1′s function to PINK1 remains unclear [71].

## 4. Concluding Remarks and Future Directions

Mitochondrial form, function, and turnover are tightly regulated by a number of intracellular signaling pathways. In particular, the mitochondria localized PKA anchoring protein AKAP1 serves vital roles in the modulation of signaling that determines mitochondrial dynamics, as well as promoting proper function to meet the changing needs of the cell. An important role of PKA at the OMM is to oppose Drp1-mediated mitochondrial fission. In this review, we have discussed how the process can be beneficial or detrimental in different diseases. The AKAP1 KO mouse model has been a versatile research tool for researchers in different fields of studies. Looking into the future, generating a tissue-specific AKAP1 KO mouse could help us to further dissect the roles of AKAP1 in different diseases. In cardiovascular diseases, hyperoxia-induced lung injury and ischemia-induced neurodegeneration, AKAP1 has been shown to support cell survival [23,24,25,27,28,50]. However, all these studies described the loss of AKAP1 as being detrimental. This raises the question of why cells limit the expression of AKAP1, and is there a disadvantage of having too much AKAP1? Will overexpression of AKAP1 provide protection or rescue any of the injuries to the tissues? In cancer, the expression level of AKAP1 seemed to be closely correlated with the metabolic program adopted by different types of cancer cell. Decreased AKAP1 expression is implicated in glycolytic metabolism dependent migrating cells found in invasive populations of breast cancer cells. Lower AKAP1 should result in reduced mitochondrial fission, resulting in more efficient mitochondrial trafficking toward the edges of the invading cells [56]. On the other hand, increased AKAP1 expression is observed in tumors of end-stage cancer patients as well as in cisplatin chemotherapy resistance [57,58,60]. Increased mitochondrial fusion enables more efficient mitochondrial respiration with OXPHOS via the electron transport chain complexes. Clearly, a more precise understanding of how AKAP1 modulates mitochondrial function in responding to upstream cellular signaling can help us better understand the metabolic reprogramming process of the cancer cells. Moreover, since AKAP1 is pro-survival, its elevation protects the cancer cells from cisplatin, presumably aiding in the development of chemotherapy resistance [60]. Collectively, these publications clearly validate further investigation into AKAP1 as a therapeutic target.
